# The peri- and intratumoral immune cell infiltrate and PD-L1 status in invasive squamous cell carcinomas of the penis

**DOI:** 10.1007/s12094-021-02694-7

**Published:** 2021-08-27

**Authors:** T. Müller, M. Demes, A. Lehn, J. Köllermann, S. Vallo, P. J. Wild, R. Winkelmann

**Affiliations:** 1 Dr. Senckenberg Institute of Pathology, Goethe University, University Hospital Frankfurt, Frankfurt am Main, Germany; 2 Institute of Biostatistics and Mathematical Modeling, Goethe University, Frankfurt am Main, Germany; 3 Institute of Virology, Goethe University, University Hospital Frankfurt, Frankfurt am Main, Germany; 4 Frankfurt Institute for Advanced Studies (FIAS), Frankfurt am Main, Germany

**Keywords:** Penile carcinomas, Tumor microenvironment, PD-L1, Survival

## Abstract

**Introduction:**

Penile carcinomas are rare tumors throughout Europe. Therefore, little attention is drawn to this disease. That makes it important to study tumor-associated key metrics and relate these to known data on penile neoplasias.

**Materials and methods:**

A cohort of 60 well-defined penile invasive carcinomas with known human papillomavirus (HPV) infection status was investigated. Data on tumor type, grading and staging were recorded. Additionally, data on the peri- and intratumoral immune cell infiltrate in a semiquanititave manner applying an HE stain were assessed.

**Results:**

Our study showed a significant correlation of immune cell infiltrate and pT stage with overall survival. Therefore, in a subset of tumors, PD-L1 staining was applied. For tumor proportion score (TPS), 26 of 30 samples (87%) were scored >0%. For the immune cell score (IC), 28 of 30 samples (93%) were defined as >0% and for CPS, 29 of 30 samples (97%) scored >0. PD-L1 expression was not associated with overall survival.

**Conclusion:**

PD-L1 is expressed in penile carcinomas, providing a rationale for targeted therapy with checkpoint inhibitors. We were able to show that immune reaction appears to be prognostically relevant. These data enhance the need for further studies on the immune cell infiltrate in penile neoplasias and show that PD-L1 expression is existent in our cohort, which may be a potential target for checkpoint inhibitor therapy.

**Supplementary Information:**

The online version contains supplementary material available at 10.1007/s12094-021-02694-7.

## Introduction

Penile carcinomas are rare tumors in Europe [1]. Usually they are detected in late stages because of prolonged interval between first recognition and consultation of a health care professional [2]. By now there are two pathways known in the development of penile carcinomas: first: penile carcinomas related to infection by human papillomavirus (HPV) with varying infection rates. Second: non-HPV-related penile carcinomas related to phimosis and chronic inflammation [3].

The infection by HPV is one of the most common sexually transmitted infections known by now. In particular HPV 16, a high-risk HPV type, is associated with the development of penile carcinomas [4]. It is known for other tumors, i.e. head and neck squamous cell carcinomas, that HPV infection in tumors is attributed to differing tumor-associated survival [5]. A surrogate marker for HPV infection is p16 expression in penile squamous cell carcinomas. Assuming that an infection with HPV leads to an exaggerated immune cell response by the host as postulated before [6], we investigated the peri- and intratumoral immune cell infiltrate in invasive penile squamous cell carcinomas. Peri- and intratumoral immune cells are investigated in a wide range of tumors with regard to recognition of prognostic factors and drug targets [7, 8]. Therefore, correlation with known survival data in our cohort was intended.

There is a study indicating that p16 expression and PD-L1 expression appear to be associated with worse clinical outcome in penile squamous cell carcinomas [9]. For non-small cell lung cancer, breast carcinoma, head and neck tumors, to only name a few, PD-L1 inhibitors are applied in tumor treatment. Detection of a positive immunohistochemical staining for PD-L1 receptor status is demanded for a variety of tumors for therapeutic purposes, i.e. head and neck squamous cell carcinomas, triple negative breast cancer, non-small cell lung cancer, melanomas and urothelial cancers. For penile squamous cell carcinomas, so far, there is no clinical demand of PD-L1 receptor status [10]. Yet, there are publications indicating PD-L1 expression in penile squamous cell carcinomas and few case reports describing the use of PD-L1 inhibitors in patients [9, 11–14]. Especially in the treatment of virally induced cancers, namely HPV infection, data on PD-L1 inhibitors are promising [15]. Data on penile carcinoma, a variably HPV attributed tumor, are sparse and clinical trials are evolving regarding checkpoint inhibitors [6, 16].

For investigation of the immune cell infiltrate we did not apply immunohistochemical markers to make our results reproducible to a wide range of pathologists regardless of their immunohistochemical repertoire. Thereafter we stained a subset of tumors with sufficient material availabe for the transmembrane programmed death ligand 1 (PD-L1, antibody ZR3). We applied statistical methods to our data to prove known tumor-associated relationships and gain advanced knowledge on our cohort of penile neoplasias in Frankfurt am Main/Germany.

## Materials and methods

### Patients and samples

A total of 60 invasive squamous cell carcinomas of the penis were identified in the archives of the Dr. Senckenberg Institute of Pathology, Frankfurt am Main. Patients received partial penectomy with or without lymphadenectomy.

Tumors were graded and characterised according to the current WHO classification and TNM system of the current version [17, 18].

The following tumor characteristics were recorded: patient age at diagnosis, TNM stage consisting of tumor stage (T), nodal stage (N), grading, tumor morphology and infiltration pattern [19]. Furthermore, p16 staining was available for all tumors and HPV status via LCD-array technique was assessed. Characteristics can be viewed in Table 1.

**Table 1:** Clinicopathological characteristics of invasive penile carcinomas

Clinicopathological characteristics of invasive penile carcinomas	Categorization	*n*	%
Age (range 41–85 years)	≤ 65 years	33	55
	> 65 years	27	45
Tumor stage	pT1a	23	38
	pT1b	7	12
	pT2	23	38
	pT3	3	5
	pT4	2	3
	pTX	2	3
Nodal status	pN0	13	22
	pN > 0	9	15
	pNX	38	63
Grading	G1	12	20
	G2	39	65
	G3	9	15
Infiltration pattern	Infiltrative pattern	38	63
	Pushing margin	22	37
Morphological tumor type	Usual	54	90
	Basaloid	6	10
HPV status	Negative	25	42
	Low risk	2	3
	High risk	31	52
	Unkown	2	3
Immune cell infiltrate	Low	4	7
	Median	24	40
	High	32	53
Immunohistochemistry (IHC)			
PD-L1 IHC (n = 30), % positive tumor cells (TPS)	TPS 0%	4	13
	TPS > 0% ≤ 10%	14	47
	TPS > 10%	12	40
PD-L1 IHC (*n* = 30), % positive immune cells (IC)	IC 0%	2	7
	IC > 0% ≤ 10%	19	63
	IC > 10%	9	30
PD-L1 IHC (*n* = 30), combined positive score (CPS)	CPS 0	1	3
	CPS > 0 ≤ 10	10	33
	CPS > 10	19	63
p16 IHC	negative	29	48
	positive	31	52

Clinical follow-up was available for all patients. Overall survival was calculated from the date of biopsy to the date of last follow-up or dead by any cause.

For our investigation, paraffin-embedded material was taken from the Dr. Senckenberg Institute of Pathology, University Hospital, Frankfurt am Main, Germany. The material was taken after diagnostics had been finished. It was double pseudonymised. Tissue samples and patient data used in this study were provided by the University Cancer Center Frankfurt (UCT). Written informed consent was obtained from all patients and the study was approved by the institutional review board of the UCT and the ethical committee at the University Hospital Frankfurt, according to the declaration of Helsinki (project-number: SUG-02-2017).

Clinicopathological characteristics are summarised in Table 1 and supplementary Table 1 for PD-L1.

### HPV status

#### DNA isolation

For HPV testing, tumor samples were macrodissected from paraffin sections of 10 µm thickness. DNA extraction was performed applying the Maxwell 16 FFPE tissue LEV DNA purification kit (Promega, Madison, WI, USA). Quantification was performed using Quantus Fluorometer (Promega, Madison, WI). DNA quality was assessed by fragment analysis (ABI Genetic Analyzer, Thermo Fisher).

#### HPV testing with LCD-array kit

Samples were subject to HPV testing by HPV 3.5 LCD-Array Kit (LCD array, Chipron, Berlin, Germany), according to manufacturers’ instructions (https://www.chipron.com). The following HPV subtypes can be detected: 6, 11, 16, 18, 31, 33, 35, 39, 42, 44, 45, 51, 52, 53, 54, 56, 58, 59, 61, 62, 66, 67, 68, 70, 72, 73, 81, 82, 83, 84, 90 and 91. A positive and a negative control were taken per run.

#### Immune cell infiltrate

**Fig. 1:**
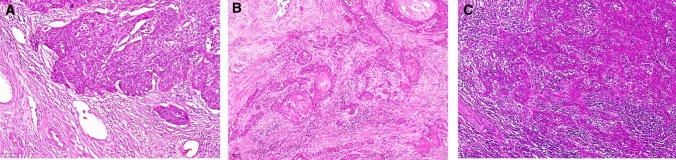
Immune cell infiltrate. **A** Few immune cells—immune cell infiltrate score 1. **B** Medium amount of immune cells—immune cell infiltrate score 2. **C** Many immune cells—immune cell infiltrate score 3

Hemotoxylin- and eosin (HE)-stained slides displaying tumor and non-tumor tissue were reviewed. Semiquantitative estimation of immune cell infiltrate was performed in peri- and intratumoral as well as non-neoplastic tissue. The peri- and intratumoral immune cells displayed immune cells of whatever kind: lymphocytes, plasma cells, granulocytes, macrophages. Scoring was performed on a one to three scale, where one means low amount of immune cell infiltrate, two means medium amount of immune cell infiltrate and three refers to many immune cells (Fig. 1). Few immune cells are only scattered cells similar to what is seen in non-neoplastic tissue. Many immune cells are dense infiltrates, possibly building germinal center-like structures. Medium amount of immune cells is in between the two beforehand described patterns. Similar evaluations can be viewed in [8].

### Antibodies

#### p16 staining

Cases were subject to p16 staining applying CINtec® Histology (Roche Diagnostics, Basel, Switzerland) antibody according to manufacturers’ instructions on Dako Autostainer link 48 (Agilent, Santa Clara, CA, USA). Block like positivity was considered positive, whereas mosaic like pattern was considered negative.

#### PD-L1 staining

Staining of PD-L1 was performed with the antibody clone ZR3 (Zeta Corporation, Arcadia, CA, USA) according to manufacturers’ instructions. In brief: Dilution: 1:200, pretreatment: Trilogy™, Cell Marque (Merck KgaA, Darmstadt, Germany), 30’ waterbath. Stainer: DAKO Autostainer Link 48, (Agilent Santa Clara, CA, USA).

We evaluated slides with more than 100 tumor cells. 30 tumors with sufficient material were subject to PD-L1 staining. The following measurements were recorded:

Tumor proportion score (TPS): percentage of viable tumor cells showing partial or complete membrane staining at any intensity, relative to viable tumor cells [20].

Immune cell score (IC): percentage of positive peri- and intratumoral immune cells located intratumoral or in peritumoral stroma at any intensity relative to tumor area [21, 22] and combined positive score (CPS): number of vital PD-L1 staining cells (tumor cells, lymphocytes, macrophages) relative to the total number of viable tumor cells, multiplied by 100 [23]. The CPS score has no dimension and is limited to 100 per definition. Granulocytes were not taken into account.

Stainings were assessed by two surgical pathologists (RW, JK) without knowledge of clinical data. For slide review a hematoxylin–eosin-stained slide was available for cross reference next to the immune stained slide to review tumor and immune cell infiltrate.

#### Statistical analysis

*P* values were regarded as descriptive means. Differences were considered statistically significant with *P* < 0.05. A statistical association between clinicopathological and other parameters was tested, using a two-sided Fisher’s exact test. Overall survival (OS) was calculated according to the Kaplan–Meier survival curves [24]. OS was measured from the start of the penectomy/resection and compared between men with or without any of the clinical, pathological, or immunohistochemical factors by two-side, d log-rank statistics [25]. Patients were censored at the time of their last clinical follow-up visit. A multivariable Cox regression model [26] was adjusted, testing the independent prognostic prevalence of the immune cell infiltrate and the pT stage. The proportionality assumption for all variables was assessed with log-negative-log survival distribution functions. For statistical calculations the programs BIAS [27], R [28], and SPSS were applied.

## Results

### PD-L1 expression in invasive penile squamous cell carcinomas

Thirty cases with sufficient material were stained with PD-L1 antibody. Patient characteristics for this cohort are summarised in supplementary Table 1.

**Fig. 2:**
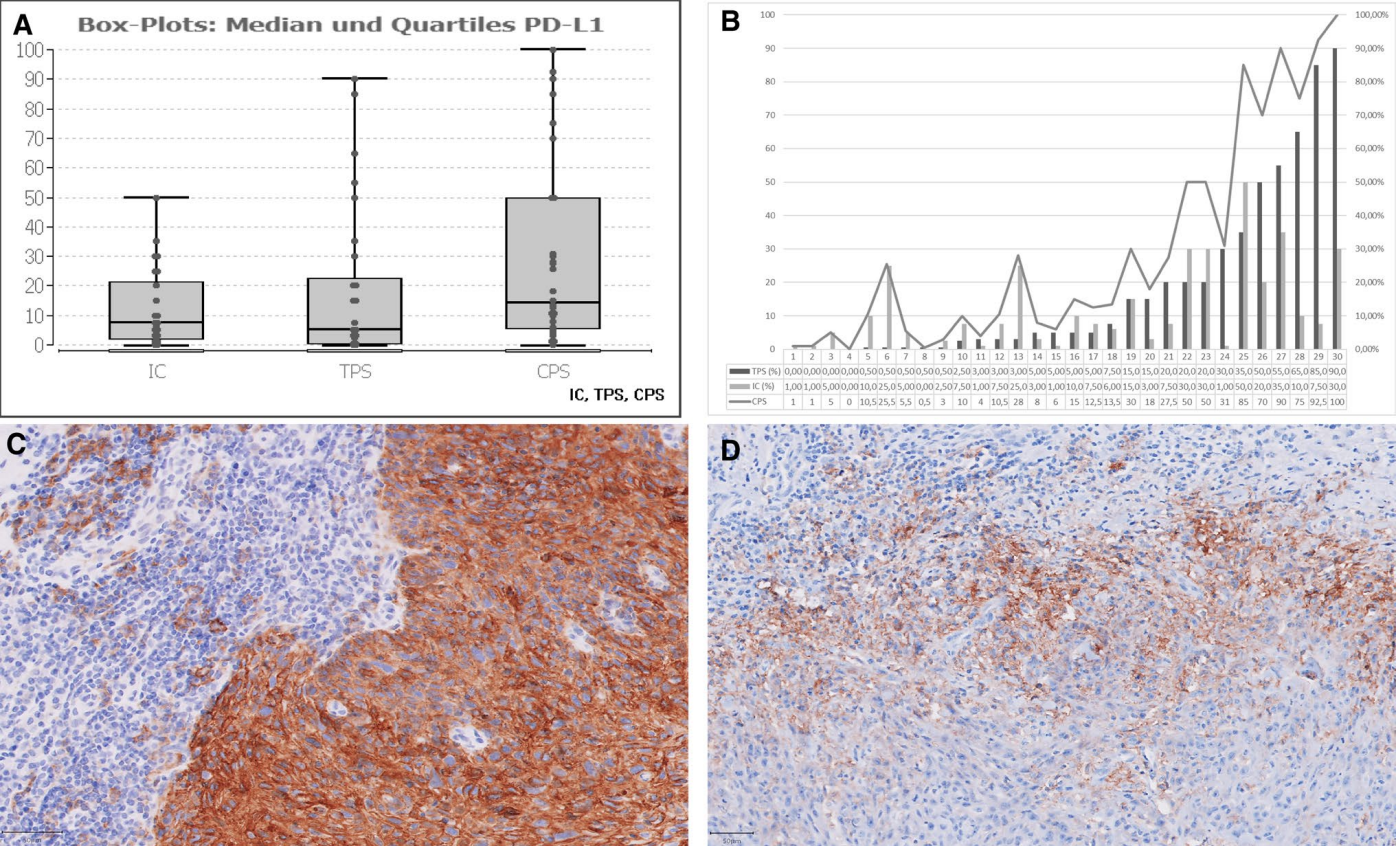
**A** Box plots with median and quartiles for IC, TPS and CPS score. **B** Cumulative representation of IC, TPS and CPS in our cohort of 30 patients (abscissa). **C** PD-L1 staining: TPS: 90%, IC: 7,5%. D: PD-L1 staining: TPS: 20%, IC: 30%

In our cohort of 30 tumors, average TPS was 18% (minimum 0%, maximum 90%, standard deviation 26%). For IC score, median was 12% (minimum 0%, maximum 50%, standard deviation 13%). Average CPS was 29 (minimum 0, maximum 100, standard deviation 36). For TPS, staining (> 0%) was shown in 26/30 cases (87%). For IC, positive immune reaction in immune cells (> 0%) was shown in 25/30 (83%) of investigated samples. Data are summarised in Fig. 2.

### The immune cell infiltrate is associated with pT stage, IC, and CPS

A total of 60 patients with penile invasive squamous cell carcinoma were studied regarding their immune cell infiltrate. Data were available for all tumors studied. Low immune cell infiltrate was noted in four cases (4/60, 7%), medium immune cell infiltrate in 24 cases (24/60, 40%) and 32 cases showed a prominent immune cell infiltrate (32/60, 53%).

**Fig. 3:**
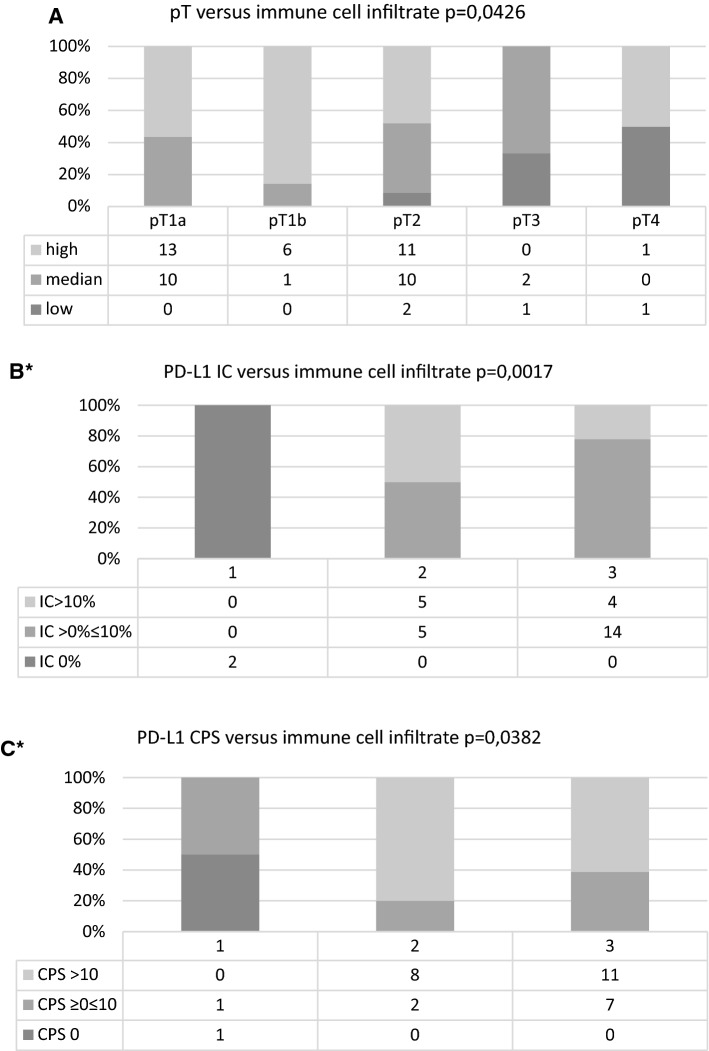
Bar charts showing pT stage, PD-L1 IC and PD-L1 CPS versus immune cell infiltrate. **A** pT versus immune cell infiltrate (ICI). B*: PD-L1 IC versus immune cell infiltrate (ICI). C*: PD-L1 CPS versus immune cell infiltrate (ICI). *1: low amount of immune cell infiltrate, 2: medium amount of immune cell infiltrate, 3: high amount of immune cell infiltrate

No significant differences for immune cell infiltrate were detected for nodal status, grading, infiltration pattern, morphological tumor type, HPV status, TPS in PD-L1 immunohistochemistry, and p16 immunohistochemistry. Immune cell infiltrate was significantly associated with tumor stage (*p* = 0.0426), IC (*p* = 0.0017), and CPS (*p* = 0.0382, Table 2, Fig. 3).

**Table 2:** Association of immune cell infiltrate with clinicopathological variables and immunohistochemistry (IHC)

		Immune cell infiltrate			
		Low	Median	High	*p*
Tumor stage	pT1a	0	10	13	0.0426
	pT1b	0	1	6	
	pT2	2	10	11	
	pT3	1	2	0	
	pT4	1	0	1	
Nodal status	pN0	0	8	5	0.0881
	pN > 0	2	2	5	
Grading	G1	1	4	7	0.8113
	G2	2	17	20	
	G3	1	3	5	
Infiltration pattern	Infiltrative pattern	2	17	19	0.5773
	Pushing margin	2	7	13	
Morphological tumor type	Usual	3	23	28	0.2098
	Basaloid	1	1	4	
HPV status	Negative	2	13	10	0.3736
	Low risk	0	1	1	
	High risk	2	9	20	
PD-L1 IHC	TPS 0%	1	0	3	0.1193
(*n* = 30)	TPS > 0% ≤ 10%	1	7	6	
	TPS > 10%	0	3	9	
PD-L1 IHC	IC 0%	2	0	0	0.0017
(*n* = 30)	IC > 0% ≤ 10%	0	5	14	
	IC > 10%	0	5	4	
PD-L1 IHC	CPS 0	1	0	0	0.0382
(*n* = 30)	CPS ≥ 0 ≤ 10	1	2	7	
	CPS > 10	0	8	11	
p16 IHC	Negative	2	14	13	0.4307
	Positive	2	10	19

### Immune cell infiltrate and pT stage in penile squamous cell carcinomas predict patient overall survival

**Fig. 4:**
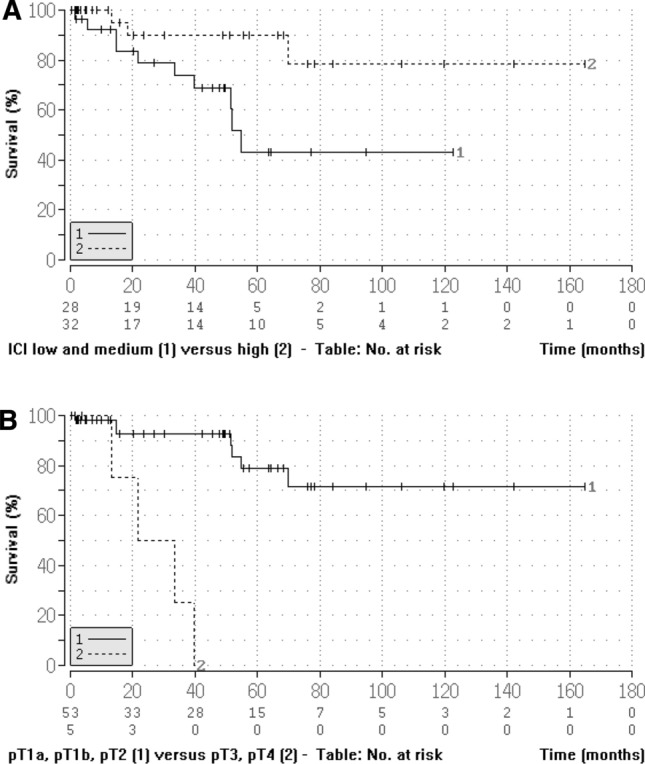
Kaplan–Meier plots for **A** immune cell infiltrate (low and medium versus high; *p* = 0.024) and **B** pT stage (pT1a, pT1, pT2 versus pT3 and pT4; *p* = 0.000). ICI, immune cell infiltrate

Univariable log-rank statistics and Cox regression analysis (Tables 3, 4, Fig. 4) revealed that the immune cell infiltrate and pT stages were associated with patient overall survival: High immune cell infiltrate and lower pT stage were associated with longer overall survival (*p* < 0.05).

**Table 3:** Univariable Cox regression analysis for invasive penile sqamous cell carcinomas (*n* = 60) regarding overall survival (log-rank test: Cox–Mantel and Peto–Pike)

Clinicopathological characteristics	Categorization	*p*	Relative hazard	95% confidence interval
age (range 41–85 years)	≤ 65 years/ > 65 years	0.483	0.679	[0.230–2.006]
Tumor stage	pT1a, pT1b, pT2/pT3, pT4	0.000	0.090	[0.034–0.238]
Tumor infiltration pattern	Infiltrative pattern/pushing margin	0.529	1.426	[0.470–4.334]
Tumor type	Usual/basaloid	N/A		
HPV status	Negative, low risk/high risk	0.374	0.607	[0.201–1.834]
Nodal status	pN0/pN > 0	0.623	0.616	[0.088–4.290]
Grading	G1, G2/G3	0.208	0.294	[0.043–2.002]
Immune cell infiltrate	Low, median/high	0.024	3.876	[1.171–12.83]
Immunohistochemistry				
PD-L1 (*n* = 30)	TPS 0%, TPS ≥ 0 ≤ 10%/TPS > 10%	N/A		
	ICS 0, ICS ≥ 0 ≤ 10%/ICS > 10%	0.632	0.565	[0.053–6.032]
	CPS 0, CPS ≥ 0 ≤ 10/CPS > 10	0.382	2.773	[0.278–2.764]
p16	Negative/positive	0.409	0.628	[0.207–1.899]

*N/A* not applicable

**Table 4:** Multivariable analysis of factors possibly influencing overall survival

	Characteristics		Global p	Hazard ratio	95% confidence interval
Univariable model					
ICI		Low, medium, high	0.036	0.246	[0.067–0.910]
pT		pT1a, pT1b, pT2, pT3, pT4	0.000	16.890	[3.694–77.218]
Multivariable raw model					
ICI	1	low and medium	0.094	0.248	[0.048–1.271]
	2	high			
pT	1	pT1a, pT1b, pT2	0.002	10.925	[2.325–51.345]
	2	pT3, pT4			
Model with dichotomised variables and interactions					
ICI	1	low and medium	0.041	0.108	[0.013–0.912]
	2	high			
pT	1	pT1a, pT1b, pT2	0.016	7.489	[1.458–38.468]
	2	pT3, pT4			
INTER		pT × ICI	0.025	59.69	[1.668–2136.074]

*ICI* immune cell infiltrate

A multiplicative term of interaction (INTER) was considered, representing an interaction between pT stage and immune cell infiltrate. The global model included immune cell infiltrate, pT stage and INTER as significant variables.

Assuming different model constructs, the following conclusion was drawn: Immune cell infiltrate and pT stage were prognostic factors for the OS in penile carcinomas (Table 4).

## Discussion

Here we show that pT stage and immune cell infiltrate are robust prognostic markers for survival in invasive squamous cell carcinomas of the penis. Low amount of immune cell infiltrate and high pT stage are indicators for poor overall survival.

There are several studies on cancer-associated tumor infiltrating lymphocytes. A review addressing several cancer types reported a statistical effect for CD3 and CD8 [29]. For example in head and neck cancer tumor infiltrating lymphocytes are associated with better prognosis, especially CD3 and CD8 positive lymphocytes, as well as improved survival data for FoxP3 positive cell infiltrates [30].

In penile carcinomas, the tumor microenvironment was also studied and identified four immunophenotypes that were related to disease specific survival and lymph node metastasis [31]. In this study, a group was identified that was characterized by a deficiency of immune cells. This subgroup was associated with higher pT stages and lymph node metastasis. These findings are similar to what was observed in this study regarding low immune cell infiltrate.

For tumors with an increased amount of peri- and intratumoral immune cells, improved overall survival was noted in our cohort. Immune cells can be associated with HPV infection status [32]. It is documented, especially in head and neck cancers, that HPV infection, evidenced by p16 immunohistochemical staining, as a surrogate marker for HPV infection, recruits immune cells [33]. Because HPV infection status was not associated with increased overall survival in our cohort, also other factors lead to increased immune cell infiltrate. This finding is in line with studies also not experiencing differences in survival concerning HPV status [32]. But the prognostic role of HPV in penile carcinomas is not yet fully elucidated and needs an increased amount of samples in further studies. Current studies are focusing on specific immune cells, mostly defined by antibodies. The complex symphony of the immune cell infiltrate is nevertheless only partly understood and needs more investigation.

High pT stage is known to be associated with decreased overall survival. Recently an update of the AJCC cancer staging guidelines for carcinomas of the penis has been adopted by the World Health Organization classification of tumors of the urinary system and male genital organs [34]. In the current edition, urethral involvement was taken out from upstaging the tumor as pT3 stage [17]. Nevertheless staging alone does not seem to reflect the tumor behaviour in its total since further metrics are sought to improve tumor categorization and estimation of the tumor behaviour [35].

For nodal status, a difference between nodal negative and nodal positive tumors was not derived in this study compared to the study conducted by Aita et al. [19].

Also, there was no statistically significant influence of the tumor invasion front in our study. Nevertheless, there are many studies concerning the invasive front of tumors. Especially in colorectal cancers, tumor cell budding is noted in the setting of aggressive tumor behaviour [36]. Also in head and neck squamous cell carcinomas, tumor cell budding and infiltration pattern were noted as diagnostic biomarkers [37]. To further elucidate the role of the invasive tumor front, uniform criteria and cut off values are needed to address the infiltration patterns to be noted. Additionally, the size of the cohort needs to be increased to address low tumor stages to generate data suitable for statistical analyses. Therefore, multicenter data are needed, especially since prediction of nodal metastasis might be more accurate than radiological findings in inguinal lymph nodes [38].

There are some publications dealing with PD-L1 status in penile squamous cell carcinomas as well as few case reports. Limiting towards those studies is the use of different antibodies for PD-L1 immunohistochemistry. Additionally, some studies consider a cut off of 5% [14] as positive and some a cut off of 1% [9] positive tumor cells. PD-L1 expression in penile cancers has been shown [39]. In some studies PD-L1 expression in penile carcinomas and a link between usual histology, lymph node status and stage was shown [13, 14, 40]. Another study showed no significant correlation between outcome and PD-L1 expression status [9]. Limiting to these studies is the small number of investigated samples. In our study we could observe a statistically significant link between IC and immune cell infiltrate as well as CPS and immune cell infiltrate. Possibly that is a bias: an increased amount of lymphocytes is possibly scored with increased IC scores, which consequently leads to increased CPS data. A link between overall survival and PD-L1 status could not be drawn in the study. That is in line with a former study [39].

## Conclusion

Invasive penile squamous cell carcinomas showed a variable amount of immune cell infiltrate. Decreased immune cell infiltrate and higher pT stages in invasive penile squamous cell carcinomas were related to shorter overall survival of patients.

**Fig. 5:**
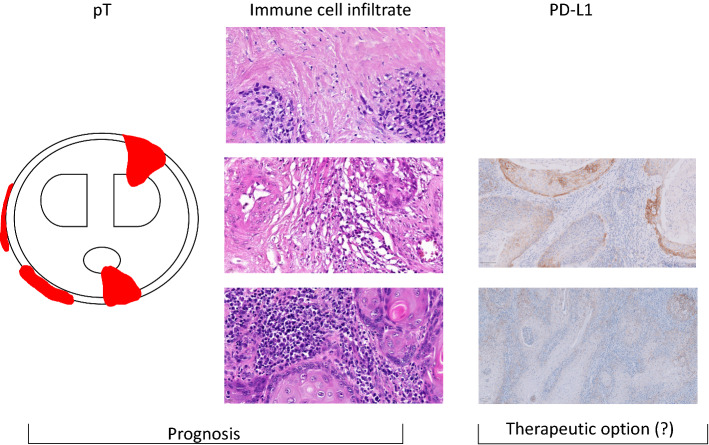
Summary: pT stage is outlined schematically with infiltration of different parts of the penis (Corpus cavernosum is diplayed in the upper part and corpus spongiosum is displayed below in an ellipse). Tumor is marked in red. This scheme is not representing complete TNM staging of penile neoplasias. Additionally the immune cell infiltrate (low, medium and high, from the top to the bottom) is presented. The left side shows different PD-L1 staining results in this cohort of penile squamous cell carcinomas

Penile squamous cell carcinomas displayed PD-L1 expression in varying proportions, creating a potential target for immunotherapy of patients with invasive squamous cell carcinomas of the penis (Fig. 5). Therefore, routine testing of PD-L1 in invasive squamous cell carcinomas of the penis should be performed for a potential off label therapy of checkpoint inhibitors.

## Supplementary Information

Below is the link to the electronic supplementary material.

Supplementary file1 (XLSX 11 KB)

## Declarations

### Availability of data and material (data transparency)

The datasets generated during and/or analysed during the current study are available from the corresponding author on reasonable request.
